# Skin infiltrating NK cells in cutaneous T-cell lymphoma are increased in number and display phenotypic alterations partially driven by the tumor

**DOI:** 10.3389/fimmu.2023.1168684

**Published:** 2023-08-25

**Authors:** Andrea Scheffschick, Julia Nenonen, Mengmeng Xiang, Anna H. Winther, Marcus Ehrström, Marie Wahren-Herlenius, Liv Eidsmo, Hanna Brauner

**Affiliations:** ^1^Division of Rheumatology, Department of Medicine, Solna and Center for Molecular Medicine, Karolinska Institutet, Stockholm, Sweden; ^2^Department of Dermatology, Karolinska University Hospital, Stockholm, Sweden; ^3^Department of Reconstructive Plastic Surgery, Karolinska University Hospital, Stockholm, Sweden; ^4^The Broegelmann Research Laboratory, Department of Clinical Science, University of Bergen, Bergen, Norway; ^5^LEO Foundation Skin Immunology Research Center, Department of Immunology and Microbiology, University of Copenhagen, Copenhagen, Denmark

**Keywords:** NK cells, CD8^+^ T cells, CTCL, mycosis fungoides, tumor environment, cutaneous lymphoma

## Abstract

Cutaneous T-cell lymphomas (CTCL) are characterized by focal infiltration of malignant T cell clones in solitary skin lesions. Many CTCL patients experience an indolent disease, but some progress to advanced disease with high fatality. We hypothesized that natural killer (NK) cells participate in local control of tumor growth in CTCL skin. Immunohistochemistry and flow cytometry analysis of the density, localization, phenotype and function of NK cells in twenty-nine fresh or formalin-fixed skin biopsies from twenty-four CTCL patients and twenty-three biopsies from twenty healthy controls highlighted higher numbers of CD56^+^CD3^-^ NK cells in CTCL skin. A reduced fraction of CTCL skin NK cells expressed the maturation marker CD57, the cytotoxic protein granzyme B and the activation marker CD69, indicating reduced tumor-killing abilities of the NK cells. Retained expression of immune checkpoint proteins or inhibitory proteins including PD1, TIM3, LAG3, CD73 and NKG2A and the activating receptors CD16 and NKp46 indicated maintained effector functions. Indeed, the capacity of NK cells to produce anti-tumor acting IFNγ upon PMA+ionomycin stimulation was similar in cells from CTCL and healthy skin. Co-cultures of primary human NK cells or the NK cell line NKL with CTCL cells resulted in reduced levels of granzyme B and CD69, indicating that close cellular interactions with CTCL cells induced the impaired functional NK cell phenotype. In conclusion, increased numbers of NK cells in CTCL skin exhibit a partially impaired phenotype in terms of activity. Enhancing NK cell activity with NK cell activating cytokines such as IL-15 or immune checkpoint blockade therefore represents a potential immunotherapeutic approach in CTCL.

## 1 Introduction

Lymphomas primarily located within the skin are rare and often arise from different stages of T-cell development (1). The majority of patients with cutaneous T-cell lymphoma (CTCL) experience an indolent disease course, but some develop an aggressive form with high fatality (2). The most common form of CTCL is mycosis fungoides (MF), which is usually restricted to the skin. In contrast, the Sezary syndrome (SS), which is the second most common form of CTCL, has malignant T cell clones present in the skin, blood and lymph nodes at diagnosis (2). In early stages of MF the disease typically presents as thin patches or more advanced thicker plaque lesions and the expected five-year survival is between 89-98%. In more advanced stages tumor development or erythroderma occur, there is a risk of spreading of the disease, and the expected five-year survival is as low as 18% (3, 4). Although hematopoietic stem cell transplantation offers a possible cure in a selected group of patients, the risks with such treatment are high and for the majority of MF patients no curative treatment is available (5).

Natural killer (NK) cells are powerful cytotoxic lymphocytes that play an important role in the defense against malignancies, particularly of hematopoietic origin, and are successfully used for cellular immunotherapy (6, 7). Upon activation, NK cells can secrete perforins, cytotoxic proteins including granzyme B or cytokines, which induces apoptosis in target cells or act in an immunomodulatory fashion (8). NK cells from blood of SS patients can lyse malignant cells (9). However, these NK cells are less potent in killing lymphoma cell than NK cells from healthy individuals, and express lower levels of the activation marker CD69 upon stimulation (10). Little is known about skin infiltrating NK cells in CTCL. NK cells are typically identified as CD56^+^CD3^-^ lymphocytes, and the presence of NK cells in CTCL skin was suggested based on the presence of CD56^+^ cells in CTCL tumors (11). However, CD56 is also expressed by the heterogeneous group of NK-like T cells, including subsets of CD4^+^ and CD8^+^ T cells, double negative (DN) T cells, γδ T cells and mucosal-associated invariant T (MAIT) cells (12, 13). Furthermore, no detailed quantification, analysis of localization and functional phenotyping of NK cells in skin from patients with CTCL has previously been reported. CD8^+^ T cells have been more studied in skin of CTCL patients. They display a cytotoxic phenotype with expression of granzyme B but also exhibit a heterogeneous expression of co-inhibitory receptors (11, 14). A detailed comparison of phenotypic changes in cytotoxic CD8^+^ T cells in CTCL *vs* healthy skin is however lacking.

We hypothesized that NK cells, similar to CD8^+^ T cells, infiltrate CTCL skin and that they have an impaired ability to reject lymphoma cells due to altered spatial distribution, phenotype or function compared to healthy skin. To investigate this possibility, we performed a detailed analysis of lymphoma-infiltrating NK cells in CTCL patient skin using immunohistochemistry, flow cytometry and performed co-culture experiments of NK cells and CTCL cell lines. In summary, we found markedly increased numbers of NK cells in fresh and formalin-fixed CTCL skin compared to healthy skin, but that a decreased proportion of NK cells in CTCL skin express the cytotoxic protein granzyme B and the activation marker CD69. In parallel, CTCL skin CD8^+^ T cells showed a similar increase in number and alterations of phenotype compared to healthy skin. The less potent NK cell phenotype found in CTCL skin was partly induced when primary healthy NK cells isolated from peripheral blood mononuclear cells (PBMC) or the NK cell line NKL were co-cultured with the human CTCL cell line HH, pointing towards a role of the lymphoma microenvironment in shaping skin NK cells in CTCL.

## 2 Materials and methods

### 2.1 CTCL patients and healthy individuals

Twenty-four CTCL patients recruited from the Department of Dermatology, Karolinska University Hospital, were included in this study and contributed with a total of twenty-nine skin biopsies. Patient characteristics are given in **Table 1**. Patients with mycosis fungoides (twenty-three patients) were clinically classified according to tumor-node-metastasis-blood (TNMB) stage, overall stage and disease activity determined by the modified severity-weighted assessment tool (mSWAT), which integrates the type of skin lesion and the affected body surface (15, 16). All TNMB and mSWAT assessments were performed by CTCL specialized medical doctors, based on direct clinical assessment, photographs and patient charts. Healthy skin samples derived from weight or breast reduction surgery were obtained from the Department of Reconstructive Plastic Surgery, Karolinska University Hospital, Solna, and Nordiska Kliniken, Stockholm, Sweden. Twenty healthy individuals, 19 females and 1 male, with an age range between 22-58 years, were included in this study and contributed a total of twenty-three skin biopsies.

**Table 1:** Characteristics of CTCL patients and their biopsies.

Pt. no.	Age	Sex	CTCL subtype	TNMB-stage^a^	Overall Stage	Type of manifestation at biopsy site	mSWAT	Treatment at the time of biopsy^b^	Fresh biopsy, size (mm)	FFPE biopsy, size (mm)
**1**	86	F	MF	T2bN0M0B0	I B	Plaque	17	Local steroid	–	4
**2a**	76	M	MF	T2bN0M0B0	I B	Plaque	35	Local steroid	–	6
**2b**						Patch			–	6
**2c**						Non-lesional			–	6
**3a**	71	F	MF	T1bN0M0B0	I A	Plaque	9	Re-PUVA	–	6
**3b**						Non-lesional			–	6
**4**	71	M	MF	T2bN0M0B0	I B	Plaque	62	PUVA	4	4
**5**	79	F	MF	T2aN0M0B1	I B	Patch	12,5	Local steroid, Alemtuzumab	4	4
**6a**	75	M	MF	T1aN0M0B0	I A	Patch	1	–	4	4
**6b**						Non-lesional		–	–	4
**7**	79	M	MF	T2bN0M0B0	I B	Plaque	20	Local steroid	6	6
**8**	48	M	MF	T2bN0M0B0	I B	Plaque	12	Acitretin	4	–
**9**	36	M	MF	T1aN0M0B0	I A	Patch	2	–	4	–
**10**	28	M	MF	T1bN0M0B0	I A	Plaque	8	Alitretinoin	4	–
**11a**	70	F	MF	T2bN0M0B0	I B	Plaque	19,5	–	3	–
**11b**						Patch		–	4	–
**12**	79	M	PTCL NOS	N/A	N/A	Tumor	21	–	4	–
**13**	59	M	MF	T2bN0MB0	IB	Plaque	15	Methotrexate	4	–
**14**	73	M	MF	T2bN0M0B0	IB	Plaque	38	Local steroid, Alitretinoin	4	–
**15**	75	M	MF	T3N0M0B0	IIB	Tumor	ND	Local steroids	–	X
**16**	61	M	MF	T1bN0M0B0	IA	Plaque	ND	–	–	X
**17**	71	M	MF	T3N1aM0B0	IIB	Tumor	ND	–	–	X
**18**	67	M	MF	T1aN0M0B0	IA	Patch	ND	–	–	X
**19**	21	M	MF, folliculotrope	T1bN0M0B0	IA	Plaque	ND	–	–	X
**20**	84	M	MF	T2bN0M0B0	IB	Plaque	ND	–	–	X
**21**	72	M	MF	T2bN0M0B0	IB	Plaque	45	–	6	–
**22**	63	M	MF	T2aN0M0B0	IB	Patch	ND	Local steroids?	–	X
**23**	83	K	MF	T2bN0M0B0	IB	Patch	ND	–	–	X
**24**	69	M	MF	T1aN0M0B0	IA	Plaque	ND	–	–	X

Pt. no. 4-7 contributed with both fresh and formalin-fixed paraffin-embedded (FFPE) biopsies. ^a^ Stage at the time of biopsy. ^b^ Local treatment at the site of the biopsy and/or systemic treatment. Fresh biopsies from patient number 7,13, 14 and 21 were included in the stimulation with PMA and Ionomycin. Fresh biopsies from the other patients were used for phenotypic characterization. CTCL, Cutaneous T-Cell Lymphoma; MF, Mycosis Fungoides; PTL NOS, Primary cutaneous Peripheral T-Cell Lymphoma Not Otherwise Specified; TNMB, Tumor-Node-Metastasis-Blood; mSWAT, Modified Severity-Weighted Assessment Tool, range 0-100 (no skin involvement – maximal skin involvement); PUVA, Psoralen and ultraviolet A; Re-PUVA, combined Retinoid and PUVA therapy. N/A, Not applicable. ND, Not determined. X, No information on biopsy size.

### 2.2 Skin biopsies

Four or 6 mm^2^ skin biopsies were taken from lesional or non-lesional skin of CTCL patients, or from healthy skin. The biopsies were either put in a sterile tube for further processing and analysis of live lymphocytes or fixed in 4% formalin for immunofluorescence staining, as further detailed below. For the isolation of lymphocytes from fresh skin biopsies, one 4 mm^2^ or half a 6 mm^2^ skin biopsy was analysed from each CTCL patient and ten 6 mm^2^ biopsies were pooled from healthy skin to isolate a sufficient number of lymphocytes.

### 2.3 Retrieval of lymphocytes from fresh skin biopsies

Skin biopsies were incubated overnight in 5 U/ml dispase (Thermo Scientific, cat. no. 17105041) at 4°C to separate epidermis from dermis. The dermis was thereafter treated with 570 U/ml collagenase III (BioNordika/Worthington, cat. no. LS004182) containing 5 µg/ml DNase I (Merck/Sigma, cat. no. 10104159001) in complete Roswell Park Memorial Institute (RPMI) medium (Gibco, cat. no. 11879020) for 90 min at 37°C followed by mechanical disaggregation with BD™ Medimachine System (cat. no. 340588). The epidermis was disrupted mechanically with scissors and pipetting to release lymphocytes. Lymphocytes from the epidermis and dermis were pooled and cultured overnight in complete RPMI medium followed by flow cytometry analysis or stimulation for functional assays (detailed below).

### 2.4 Retrieval of lymphocytes from blood

PBMCs were isolated from buffy coats by Ficoll (Merck/Sigma, cat. no. F4375) density gradient centrifugation and stored at -80°C for short term storage and in liquid nitrogen for long-term storage until analysis. Primary NK cells were isolated from PBMCs by negative selection with magnetic beads (Miltenyi Biotec, NK Cell Isolation Kit, cat. no. 130-092-657) according to the manufacturer’s instructions.

### 2.5 Flow cytometry analysis

Skin and blood derived lymphocytes were first stained with live/dead dye (LIVE/DEAD™ Fixable Aqua Dead, Invitrogen, cat. no. L34966) and thereafter stained with fluorescent-conjugated monoclonal antibodies. Antibodies used for flow cytometry analysis are displayed in **Supplementary Table S1**. The antibodies were diluted in PBS with 1% FCS and staining was performed for 30 min at 4°C. The cells were thereafter fixed and permeabilized for intracellular staining using a BD Cytofix/Cytoperm™ Kit (BD Biosciences, cat. no. 554714). For functional phenotyping experiments, lymphocytes from skin were treated for 4 h with phorbol 12-myristate 13-acetate (PMA, 10 ng/ml, Sigma-Aldrich, cat. no. 524400) and ionomycin (1 µM, Sigma-Aldrich, cat. no. 407950) followed by addition of Brefeldin A (5 µg/ml, Sigma-Aldrich, cat. no. B6542) after 1 h. Live/dead dye, cell membrane and intracellular stainings were thereafter performed as described above. For *in vitro* co-culture and conditioned media experiments, cells were co-cultured or stimulated as described in the Cell culture section (paragraph 2.7), followed by addition of Brefeldin A after 1 h. Cells were stained with live/dead dye, cell membrane and intracellular antibodies as described above. Cells were analyzed on an LSR Fortessa™ flow cytometer using the BD FACS Diva™ software (BD Biosciences Immunocytometry Systems). All samples were analyzed without delay to ensure optimal technical quality and appropriate controls were performed, including “fluorescence minus one” control samples. Quality control of the flow cytometer’s performance and CV values were monitored on a day-to-day basis using CS&T beads (BD Biosciences, cat. no. 656505).

### 2.6 Immunofluorescence

Skin biopsies were fixed for 24-49 h in 4% formalin and then moved to 70% ethanol, dehydrated and embedded in paraffin. The tissue was cut into 3.5 - 4 µm sections, placed on Superfrost plus glass slides (Fisher Scientific, cat. no. 12-550-15) and incubated over-night at 37°C to ensure tissue attachment. Sections were stored at -20°C until staining. Formalin-fixed and paraffin-embedded skin sections were stained with the primary and secondary antibodies listed in **Supplementary Table S1**. To visualize CD56, a tyramide signal amplification (TSA) Plus Fluorescein kit (Akoya, cat. no. NEL741E001KT) was used according to the manufacturer´s instructions. Nuclei counterstain was performed using 4′,6-diamidino-2-phenylindole DAPI (Invitrogen, cat. no. D1306) and embedding was performed using ProLong™ Gold Antifade Mountant (Invitrogen, cat. no. P10144). The immunofluorescence stainings were assessed using either a Zeiss AxioScan.Z1 slide scanner or a Zeiss LSM700 microscope and Zen lite software. Counting of NK cells and CD8^+^ T cells was performed manually using ImageJ and Zen lite software in a representative 20x tile scan. Analyzed tissue area was measured using ImageJ and ranged between 0,77 mm^2^ – 2,63 mm^2^. Cell numbers were normalized to analyzed tissue area and reported as cells/mm^2^.

### 2.7 Cell culture

The human malignant CD4^+^ CTCL cell line HH from CTCL patient blood was purchased from ATCC (cat. no. CRL­2105) and the human non-malignant CD4^+^ T cell line MyLa from CTCL patient skin was purchased from Sigma-Aldrich (cat. no. 95051032-1VL). The human NK cell line NKL has previously been described and was a gift from Dr. M. J. Robertson (Indiana University School of Medicine, Indianapolis, IN) (17). All cell lines were maintained in RPMI 1640 medium with 10% FCS (Sigma, cat. no. F7524-500ML), 2 mM L-Glutamine (Sigma, cat. no. G7513), 100 IU/ml penicillin and 100 µg/ml streptomycin (Sigma, cat. no. P4333) (further referred to as complete RPMI medium) in a humidified atmosphere with 5% CO_2_ at 37°C. The NKL culture was supplemented with IL-2 (100 U/ml, PeproTech, cat. no. 200-02).

Co-culture experiments were performed as follows: The freshly isolated primary NK cells from PBMC were cultured in complete RPMI 1640 medium supplemented with IL-2 (1000 U/ml) for 48 h before the experiments. For co-culture experiments, NKL or primary NK cells were co-cultured in a 1:1, 1:2 and 1:5 ratio with HH or MyLa cell lines for 6 hours in complete RPMI medium supplemented with IL-2 (100 U/ml) at 37°C. Co-culture of NK cells with HH and MyLa cells in a 1:5 ratio showed the strongest effect and was used for experiments (data not shown). Following 6 hours of co-culture, the NK cell phenotype was investigated using flow cytometry. To distinguish NKL cells and primary NK cells from T cells in the co-culture cell suspension, the gating strategies presented in **Supplementary Figures S1**, **S2** were used.

Experiments with conditioned medium were performed as follows: The freshly isolated primary NK cells from PBMC were cultured in complete RPMI 1640 medium supplemented with IL-2 (1000 U/ml) for 48 h before the experiments. For stimulation of NKL or primary NK cells with conditioned medium of HH and MyLa cells, HH or MyLa cells were first grown for 24 h and the conditioned medium was collected after centrifugation at 200 g for 5 min to pellet the cells. NKL cells or primary NK cells were thereafter stimulated in a 1:2 ratio of conditioned medium and complete RPMI medium supplemented with 100 U/ml IL-2 or cultured with medium with 100 U/ml IL-2 only followed by flow cytometry analysis.

### 2.8 Ethical considerations

All experiments for this study were approved by the regional ethics committee Stockholm and the Code of Ethics of the World Medical Association (Declaration of Helsinki) for human samples was followed. All participants gave written consent.

### 2.9 Statistical analysis

Statistical analysis was performed using the Mann-Whitney U-test, the Wilcoxon test, ordinary one-way ANOVA with Tukey’s multiple comparison test, Kruskal-Wallis test with Dunn’s multiple comparison test or Friedman test with Dunn´s multiple comparison test as appropriate. * = p < 0.05, ** = p < 0.01, *** = p < 0.001. Non-parametric correlation analysis using Spearman correlation coefficient was performed to assess associations between clinical parameters (mSWAT for disease activity), Visual Analogue Scale (VAS) for itch, lactate dehydrogenase (LD), erythrocyte sedimentation rate (ESR), C-reactive protein (CRP), eosinophils, neutrophils, hemoglobin, leukocytes, lymphocytes) and NK cell numbers, NK cell percentages or phenotypical marker.

### 2.10 Data availability

Data were generated by the authors and are available on request.

## 3 Results

### 3.1 NK cells infiltrate CTCL skin

**Figure 1:**
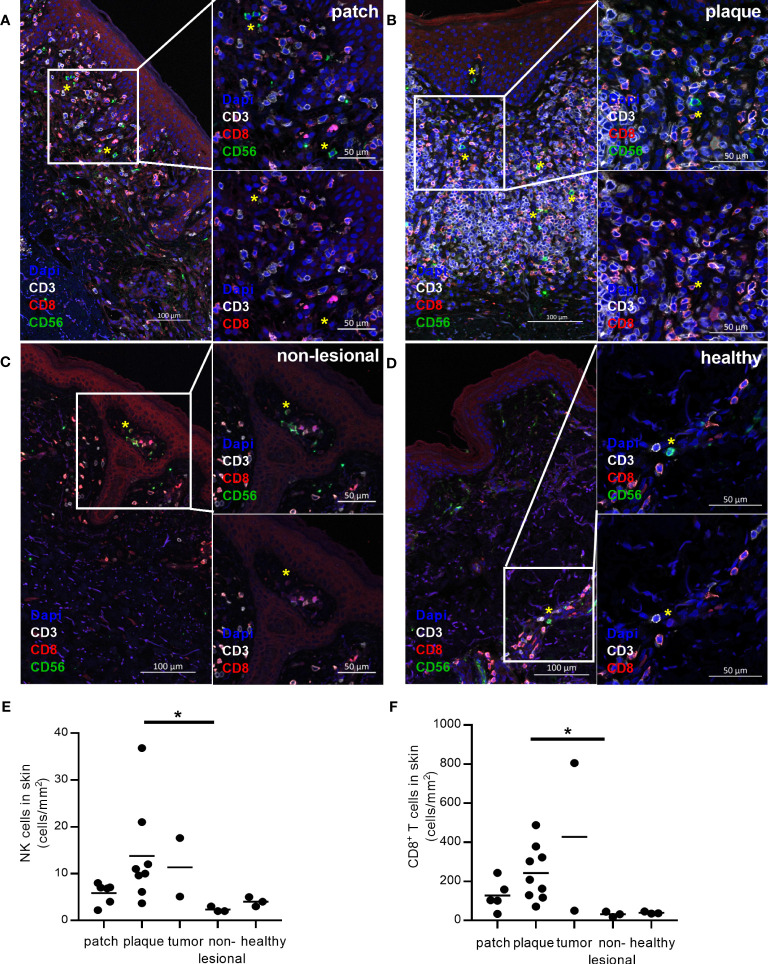
Infiltration of NK cells in formalin-fixed CTCL skin compared to healthy skin and non-lesional CTCL skin. CD3, CD8 and CD56 co-staining was performed to identify CD56^+^CD3^-^ NK cells (green cells, marked with yellow *) and CD3^+^CD8^+^ T cells (red cells) in formalin-fixed and paraffin-embedded skin. NK cells and CD8^+^ T cells were identified in **(A)** patch, **(B)** plaque, and **(C)** non-lesional skin from CTCL patients and **(D)** skin from healthy controls. Numbers of **(E)** NK cells and **(F)** CD8^+^ T cells identified in skin per mm^2^ tissue section. Data from formalin-fixed skin biopsies of three healthy controls and sixteen CTCL patients (contributing with in total twenty skin biopsies), including six patch lesions, nine plaque lesions, two tumor lesions, and three biopsies from non-lesional skin. For CD8^+^ T cell counting, one patient was excluded from the analysis as the malignant cells of this patient were CD8^+^. For NK cell counting, one patient was excluded from the analysis due to technical difficulties to discriminate between NK and NK-like T cells. Kruskal-Wallis tests with Dunn´s multiple comparison tests were performed. * = p < 0.05.

To identify CD56^+^CD3^-^ NK cells and CD3^+^CD8^+^ T cells in formalin-fixed CTCL skin biopsies, we applied an immunofluorescence triple staining of CD3, CD56 and CD8. Twenty skin biopsies from sixteen CTCL patients and three healthy skin biopsies were analysed and all contained varying numbers of skin infiltrating NK cells (representative images in **Figures 1A–D** and quantification in **Figure 1E**, patient information in **Table 1**). NK cell numbers were significantly higher in plaque CTCL skin as compared to non-lesional CTCL skin (**Figure 1E**). Interestingly, NK cells were detected throughout all biopsies, indicating their capacity to infiltrate into the lymphoma. In line with previous reports, CD8^+^ T cells were present in higher numbers in CTCL skin compared to non-lesional skin (**Figure 1F**) (11).

### 3.2 The CTCL skin NK cell population is enriched for the CD56^bright^ subset

**Figure 2:**
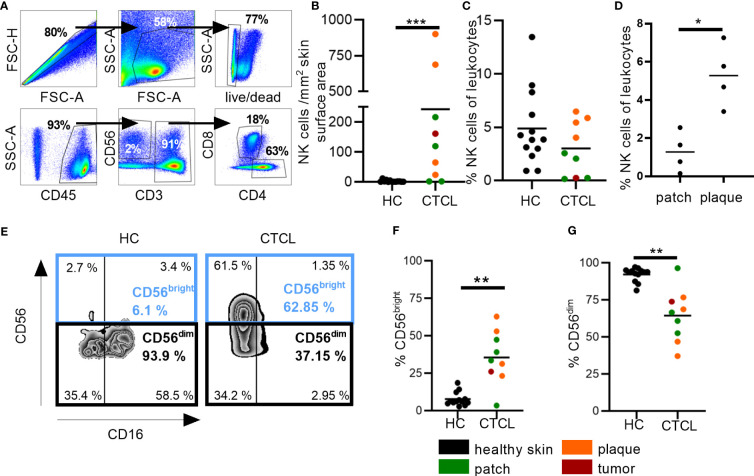
Increased numbers of NK cells in fresh CTCL skin and enrichment of the CD56^bright^ NK cell subset. Flow cytometric analysis for investigating numbers and percentages of NK cells and NK cell subsets in CTCL skin and healthy skin. **(A)** Cells were gated on singlet lymphocytes, live cells, CD45^+^ cells and thereafter CD56^+^CD3^-^ NK cells and CD3^+^CD8^+^ T cells. **(B)** Numbers of NK cells per mm^2^ skin surface area are presented. **(C)** Percentages of NK cells of total leukocytes are given. **(D)** Percentages of NK cells of total leukocytes in patch compared to plaque skin are given. **(E)** Identification of CD56^bright^ and CD56^dim^ NK cells in healthy control (HC) and CTCL skin using the markers CD56 and CD16. **(F)** Percentages of CD56^bright^ and **(G)** CD56^dim^ NK cells are given. Data from fresh skin biopsies of thirteen healthy controls and eight CTCL patients (one patient contributed with two biopsies, one from a patch and one from a plaque lesion). Mann-Whitney U-tests were performed. * = p-value < 0.05, ** = p-value < 0.01, *** = p-value < 0.001.

We thereafter verified the presence of NK cells in skin by flow cytometry (**Table 1**; **Figure 2A**). Markedly increased numbers of NK cells, as well as CD8^+^ T cells, were identified in CTCL skin as compared to healthy skin (**Figure 2B**; **Supplementary Figure S3A**). The absolute numbers of NK cells and CD8^+^ T cells were not significantly different in patch *vs* plaque lesions of CTCL patients (**Supplementary Figure S3B, C**). The frequency of NK cells of total leukocytes was similar in CTCL skin and healthy skin, but markedly higher in plaque compared to patch lesions in the patients (**Figures 2C, D**). In contrast, the frequency of CD8^+^ T cells of total leukocytes was lower in CTCL skin compared to healthy skin and not significantly different in patch *vs* plaque lesions (**Supplementary Figures S3D, E**).

Next, we identified the frequencies of the CD56^bright^CD16^dim/-^ (so called CD56^bright^) and CD56^dim^CD16^bright/-^ (so called CD56^dim^) subsets of NK cells (**Figure 2E**). Most NK cells in healthy skin and CTCL skin belonged to the CD56^dim^ subset, which has strong cytotoxic potential. However, there was a marked enrichment of the cytokine secreting CD56^bright^ NK cell subset in CTCL skin (**Figures 2F, G**).

### 3.3 NK cells in CTCL skin display a less activated phenotype

**Figure 3:**
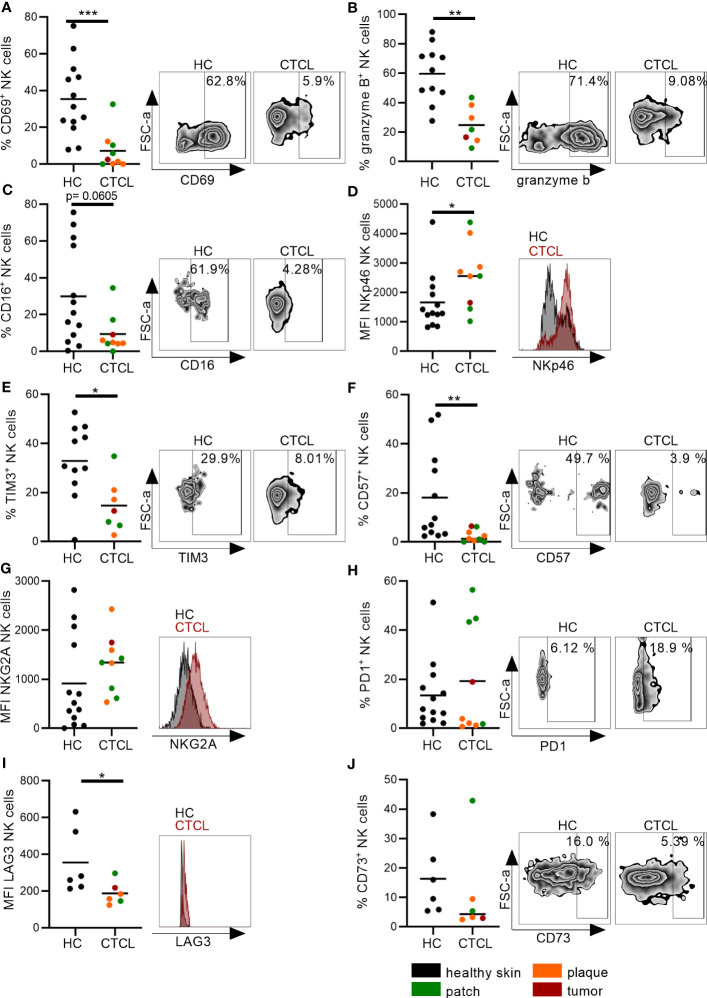
Distinct phenotype of CTCL skin NK cells indicates reduced activation and maturation. NK cells from CTCL and healthy skin were characterized by **(A)** the early activation marker CD69, **(B)** the cytotoxic protein granzyme B, **(C)** the activating receptor CD16, **(D)** the activating receptor NKp46, **(E)** the immune checkpoint protein TIM3, **(F)** the maturation marker CD57, **(G)** the inhibitory receptor NKG2A and **(H–J)** the immune checkpoint proteins PD1, LAG3 and CD73. Data from fresh skin biopsies of thirteen healthy controls and eight CTCL patients (one patient contributed with two biopsies, one from a patch and one from a plaque lesion). Some phenotypic markers were not analyzed in all biopsies. Cells were gated on singlet lymphocytes, live cells, CD45^+^ cells and thereafter CD56^+^CD3^-^ NK cells. For gating strategy see **Figure 2A**. Mann-Whitney U-tests were performed. * = p-value < 0.05, ** = p-value < 0.01, *** = p-value < 0.001.

To characterize the NK cells of CTCL skin further, we performed a detailed phenotypic analysis. Although the phenotype of CTCL skin CD8^+^ T cells has been investigated in previous studies with regard to granzyme B levels, immune check point proteins and inhibitory receptors, a thorough comparison to healthy skin CD8^+^ T cells and to CTCL skin NK cells was lacking. We found that significantly fewer NK cells and CD8^+^ T cells expressed the activation marker CD69 and the cytotoxic protein granzyme B in CTCL skin compared to healthy skin (**Figures 3A, B**; **Supplementary Figures S4A, B**). Furthermore, the frequency of CTCL skin NK cells that expressed CD16, crucial for eliciting antibody-dependent cellular cytotoxicity (ADCC), was unaltered but had a tendency towards downregulation (p=0.061) (**Figure 3C**). The activating receptor NKp46 was expressed at higher levels on CTCL skin NK cells compared to healthy skin (**Figure 3D**) and fewer CTCL skin NK cells and CD8^+^ T cells expressed the inhibitory surface receptor TIM3 (**Figure 3E**; **Supplementary Figure S4E**). A smaller fraction of the CTCL skin NK cells expressed the maturation marker CD57 (**Figure 3F**), while there was no difference in CD57 expression between CD8^+^ T cells from CTCL and healthy skin (**Supplementary Figure S4F**). The expression of the inhibitory receptor NKG2A and the checkpoint proteins PD1 and CD73 were similar on both NK cells and CD8^+^ T cells in CTCL and healthy skin, but the immune checkpoint protein LAG3 was downregulated on CTCL NK cells compared to healthy NK cells (**Figures 3G–J**; **Supplementary Figures S4G–J**). To identify differences in NK cell phenotypes in patch *vs* plaque manifestations of CTCL, patients were grouped depending on the type of skin lesion (**Figure 3**). No significant differences were however observed, possibly due to the small sample size. Correlation analysis between NK cell numbers, percentages or phenotypic markers and clinical laboratory parameters revealed strong positive correlations between serum levels of lactate dehydrogenase (LD) and % CD16^+^ NK cells (r=0.79, p=0.048) and for blood neutrophils and % CD57^+^ NK cells (r=0.78, p=0.028). Strong inverted correlations were observed for blood eosinophils and % TIM3^+^ NK cells (r=-0.88, p=0.033), for blood hemoglobin and % CD69^+^ NK cells (r=-0.75, P=0.037) as well as for blood lymphocytes and % CD16^+^ NK cells (r=-0.73, p=0.046). No significant correlations were found between disease activity as measured by mSWAT or itch measured on a visual analogue scale with NK cell numbers, percentages or phenotypic markers (data not shown). For CD8^+^ T cell phenotype in patch *vs* plaque however, there was a tendency to higher proportions of CD69 positive CD8^+^ T cells in patch compared to the more advanced plaque lesions (p=0.057) (**Supplementary Figure S4A**). Taken together, CTCL skin NK cells, similar to CD8^+^ T cells, express the activation marker CD69 and the cytotoxic protein granzyme B to a lesser degree than healthy skin NK cells and CD8^+^ T cells, potentially indicating a less active phenotype. However, elevated levels of NKp46 on CTCL skin NK cells might indicate that NK cells can still be activated. The expression of classical immune checkpoint proteins and inhibitory receptors was however largely unaltered. The phenotype of healthy and CTCL skin NK cells and CD8^+^ T cells is summarized in **Table 2**.

**Table 2:** Summary of phenotypic markers on NK cells and CD8^+^ T cells in healthy and CTCL skin analyzed by flow cytometry.

phenotypic marker	NK cells		p-value	CD8^+^ T cells		p-value
	HC (mean ± SD)	CTCL (mean ± SD)		HC (mean ± SD)	CTCL (mean ± SD)	
**CD69**	35,4 ± 20,37	7,3 ± 10,55	0.001	46,4 ± 19,27	11,0 ± 13,43	0.001
**granzyme B**	59,7 ± 19,26	24,7 ± 12,85	0.001	42,0 ± 16,97	14,5 ± 10,89	0.002
**CD16**	30,0 ± 26,75	9,4 ± 10,54	0.061	n.a.	n.a.	n.a.
**PDL1**	n.a.	n.a.	n.a.	121,8 ± 186,93	398,5 ± 287,78	0.200
**NKp46**	1662,8 ± 964,34	2578,0 ± 1119,47	0.025	328,1 ± 196,64	198,2 ± 268,62	0.204
**TIM3**	32,9 ± 15,06	14,7 ± 10,89	0.020	70,3 ± 34,39	21,2 ± 31,31	0.003
**CD57**	17,6 ± 17,87	2,4 ± 2,53	0.003	13,4 ± 6,73	15,0 ± 8,77	0.832
**NKG2A**	914,3 ± 958,95	1314,6 ± 599,17	0.144	1446,4 ± 1318,22	2349,2 ± 1057,42	0.123
**PD1**	13,4 ± 13,71	19,2 ± 22,73	0.695	54,0 ± 17,97	55,8 ± 20,84	0.845
**LAG3**	355,8 ± 176,88	187,8 ± 62,54	0.041	365,9 ± 182,23	285,8 ± 41,15	0.132
**CD73**	16,3 ± 12,65	11,1 ± 15,81	0.132	48,0 ± 23,49	22,2 ± 16,51	0.065

Blue represents data in %. Green represents MFI data. HC, healthy control; CTCL, cutaneous T-cell lymphoma; n.a., not available.

### 3.4 CTCL skin NK cells are able to produce IFNγ upon stimulation with PMA and ionomycin

**Figure 4:**
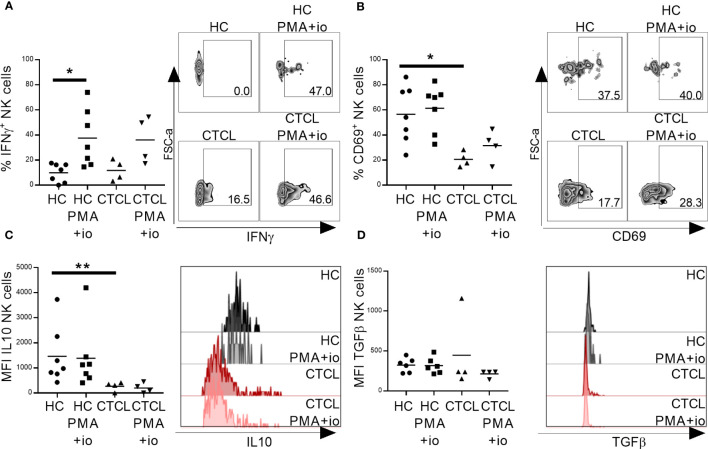
Primary CTCL skin NK cells are able to produce IFNγ upon *in vitro* activation with PMA and ionomycin. Total lymphocytes extracted from CTCL and healthy skin were stimulated for 4 h with PMA and ionomycin followed by flow cytometric analysis of NK cells for IFNγ **(A)**, CD69 **(B)**, IL-10 **(C)** and TGFβ **(D)**. Data from fresh skin biopsies of seven healthy controls and four CTCL patients. Cells were gated on lymphocytes, single cells, live cells, CD45^+^ cells and thereafter CD56^+^CD3^-^ NK cells. For gating strategy see **Supplementary Figure S6**. Mann-Whitney U-tests were performed to assess differences between healthy skin and CTCL skin. Wilcoxon tests were performed to assess differences between unstimulated and stimulated samples. * = p-value < 0.05, ** = p-value < 0.01.

Based on their less activated phenotype, we speculated that CTCL skin NK cells would be functionally impaired. To test this hypothesis, we investigated their capacity to produce cytokines crucial for anti-tumor immune responses upon PMA and ionomycin stimulation *in vitro.* Interestingly, CTCL NK cells, as well as CTCL CD8^+^ T cells, were able to produce pro-inflammatory IFNγ to the same level as cells derived from healthy skin, indicating normal cytokine secreting capacity. The IFNγ induction was however not significant in CTCL samples, probably due to the small sample size (**Figure 4A**; **Supplementary Figure 5A**, representative gating strategy is shown in **Supplementary Figure S6**). In line with this, the stimulation resulted in upregulation of the early activation marker CD69, although not significantly in this small cohort (**Figure 4B**; **Supplementary Figure 5B**). Anti-inflammatory cytokines IL-10 and TGFβ were not induced upon stimulation. Notably though, IL-10 levels in unstimulated healthy NK and CD8^+^ T cells were higher compared to CTCL NK and CD8^+^ T cells, although only significantly for NK cells, while TGFβ was only significantly higher in unstimulated healthy CD8^+^ T cells compared to CTCL CD8^+^ T cells (**Figures 4C, D**; **Supplementary Figures S5C, D**).

### 3.5 CTCL cell line HH causes functional phenotypic changes in NK cells in a cell-cell contact dependent manner

**Figure 5:**
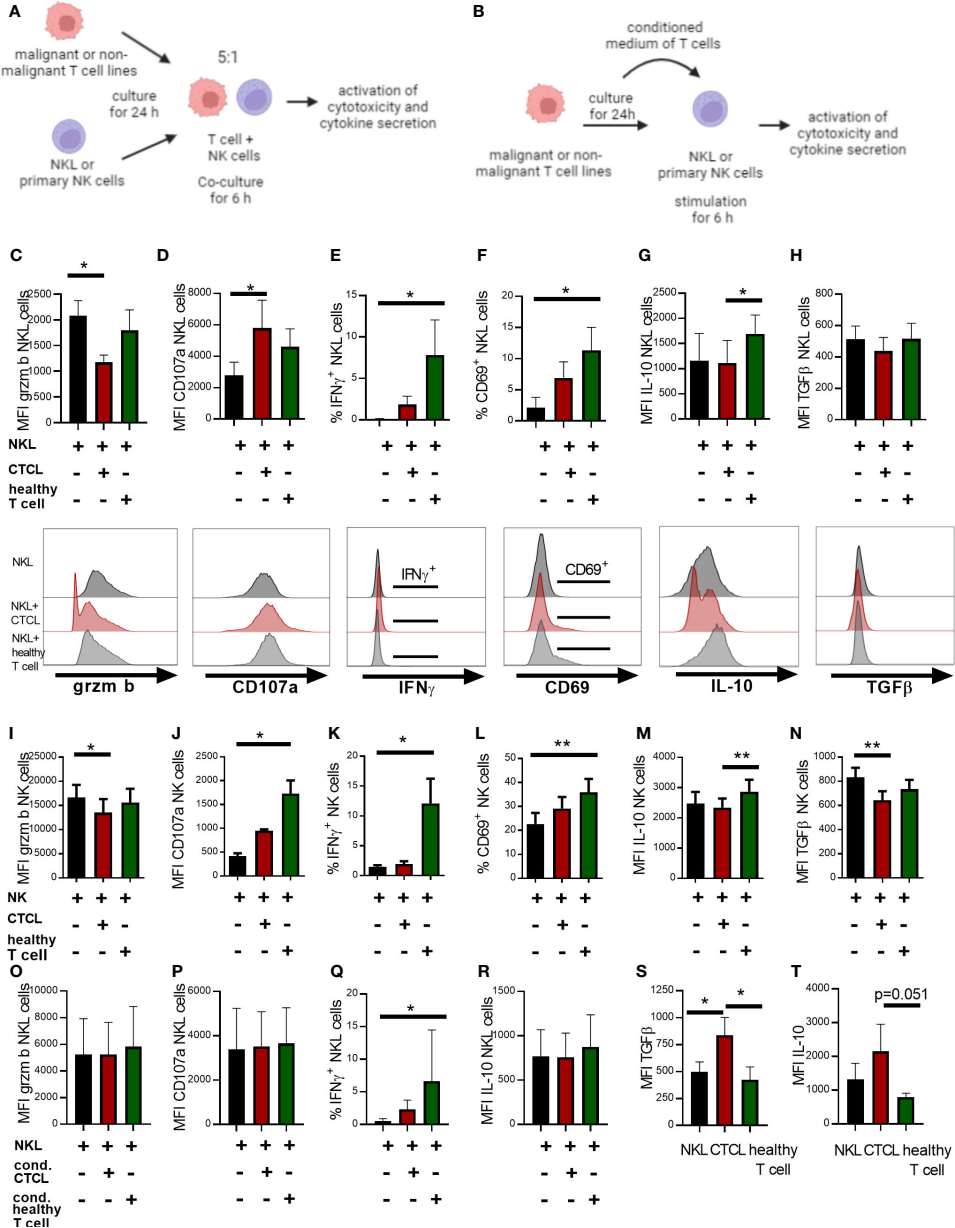
Co-culture of NK cells with the HH CTCL cell line reduces granzyme B positive NK cell proportions in a cell-contact dependent manner and fails to fully upregulate IFNγ and CD69, compared to non-malignant T cell line MyLa CD4. Illustrative images showing the experimental set up of NK cell **(A)** co-culture with malignant or non-malignant T cells lines or **(B)** stimulated with their conditioned medium. The NKL cell line was co-cultured for 6 h in a ratio of 1:5 with the CTCL cell line HH and non-malignant T cell line MyLa CD4 followed by flow cytometry analysis of NKL cell phenotype for granzyme B **(C)**, CD107a **(D)**, IFNγ **(E)**, IL-10 **(F)**, TGFβ **(G)** and activation marker CD69 **(H)**. NK cells isolated from PBMC were co-cultured for 6 h in a ratio of 1:5 with the CTCL cell line HH and non-malignant T cell line MyLa CD4 followed by flow cytometry analysis of NK cell phenotype for granzyme B **(I)**, CD107a **(J)**, IFNγ **(K)**, IL-10 **(L)**, TGFβ **(M)** and activation marker CD69 **(N)**. NKL cells were stimulated for 6 h with conditioned medium of the CTCL cell line HH or non-malignant T cell line MyLa CD4, followed by flow cytometric analysis of NKL cell phenotype with regard to granzyme B **(O)**, CD107a **(P)**, IFNγ **(Q)**, and IL-10 **(R)**. Analysis of TGFβ protein **(S)** and IL-10 protein **(T)** in unstimulated NKL cells, the advanced CTCL cell line and the non-malignant T cell line. Data represent results from three to six independent experiments of the NKL cell co-cultures (n=4), the primary NK cell co-cultures (n=4-6) and the analysis of TGFβ and IL-10 in unstimulated cells (n=3). Gating strategy for the co-culture experiments is shown in **Supplementary Figures S1**, **S2**. Friedman tests with Dunn´s multiple comparison tests were performed for the co-culture experiments and the stimulations with conditioned media. Ordinary one-way ANOVA with Tukey´s multiple comparison tests were performed for comparing TGFβ and IL-10 levels in the unstimulated cells. * = p-value < 0.05, ** = p-value < 0.01. Error bars indicate SD. Images in **(A, B)** have been created with BioRender.com.

We lastly speculated that the observed alterations in NK cell phenotype in CTCL skin arise due to interactions with the lymphoma. To test this possibility, we co-cultured NK cells with the malignant CD4^+^ CTCL cell line HH or the non-malignant CD4^+^ T cell line MyLa derived from CTCL patient skin, or stimulated NK cells with conditioned media of these cells. Thereafter, we investigated the effect on key NK cell functional phenotypes identified in the fresh primary skin biopsies from CTCL skin (**Figures 3**, **4**) by flow cytometry. We assessed the early activation marker CD69, the cytotoxic protein granzyme B complemented with the surrogate marker for cytotoxicity CD107a, the anti-inflammatory cytokines IL-10 and TGFβ, and in addition the pro-inflammatory cytokine IFNγ (the experimental set up is schematically presented in **Figures 5A, B**). NK cells (NKL cells or primary NK cells from healthy PBMC) cultured with malignant HH cells showed decreased levels of granzyme B and increased levels of CD107a compared to unstimulated NK cells, suggesting that granzyme B was released from NK cells and that NK cell killing was induced (**Figures 5C, D, I, J**). In contrast, NK cells co-cultured with the non-malignant MyLa cells showed no reduction in granzyme B but an induction of CD107a (**Figures 5C, D, I, J**). Interestingly, NK cells co-cultured with the non-malignant MyLa cells showed a significant induction of IFNγ and CD69, indicating NK cell activation, while IFNγ and CD69 levels in NK cells co-cultured with the malignant HH cells remained low (**Figures 5E, F, K, L**; **Supplementary Figures S7A, B**). Furthermore, IL-10 was upregulated in NK cells co-cultured with the non-malignant MyLa compared to NK cells co-cultured with the malignant HH cells (**Figures 5G, M**), while TGFβ was not induced (**Figures 5H, N**).

We next stimulated NKL cells with conditioned medium of malignant HH cells, but observed no impact on granzyme B and CD107a levels, indicating that close cell contact is required for these phenotypic changes to occur (**Figures 5O, P**). IFNγ was however significantly upregulated in NKL cells stimulated with conditioned medium from the non-malignant MyLa cells, but not with conditioned media from the malignant HH cells (**Figure 5Q**), while IL-10 was not significantly changed (**Figure 5R**). A hallmark of advanced CTCL is increased levels of IL-10 and TGFβ (18). The malignant HH cells showed higher levels of TGFβ and IL-10 (**Figures 5S, T**), although not significantly for IL-10 (p=0.051), compared to non-malignant MyLa cells, which could contribute to the impaired activation of NK cells. In summary, co-culture of NK cells with malignant CTCL cells partially induces the phenotype observed in fresh CTCL skin NK cells with reduced CD69 and granzyme B levels, suggesting that close contact with malignant cells is required for this phenotype.

## 4 Discussion

NK cells and CD8^+^ T cells have powerful anti-lymphoma effector function and immunotherapy that increases their activity in cancer leads to increased patient survival (7, 19). Infiltration of NK cells into solid tumors furthermore correlates with longer overall survival of patients in different cancer types (20). Within this study, we identified NK cells in CTCL skin and investigated their phenotype and function compared to healthy skin NK cells and to CTCL CD8^+^ T cells. Overall, the numbers of NK cells and CD8^+^ T cells in CTCL skin were increased as compared to healthy skin. This could be due to infiltration of immune cells from the circulation or expansion of skin-resident cells. We speculated that NK cells would have difficulties to penetrate into the lymphoma tissue, similar to what has been shown for several other solid tumors (21, 22). However, we could identify NK cells throughout the whole lymphoma tissue. The absolute number of NK cells, but not CD8^+^ T cells, had a tendency towards increase in more advanced plaque as compared to early patch lesions, although the patient number was too small to make firm conclusions. The percentages of CD8^+^ T cells in CTCL skin were previously shown to decrease in the CTCL tumors as compared to plaques (11), possibly due to a strong expansion of tumor cells. Hence, it was suggested that high percentages of CD8^+^ T cells in CTCL skin are a good prognostic factor. In line with this, we observed a tendency to reduced CD8^+^ T cell percentages in plaques compared to patches. Since NK cell percentages increased in plaques compared to patches it could be proposed that NK cells locally in CTCL skin lesions are more important for the disease outcome than CD8^+^ T cells in more advanced disease manifestations.

In line with previous findings on CD8^+^ T cells (11), we identified a decreased proportion of both NK cells and CD8^+^ T cells expressing granzyme B and CD69 in CTCL skin. A reduction of granzyme B in cytotoxic cells could indicate that target cell killing has occurred, leading to depletion of cytotoxic granules. Alternatively, cytotoxic cells may fail to express granzyme B in the tumor environment, resulting in reduced NK cell and CD8^+^ T cell driven tumor cell killing. Decreased levels of granzyme B in NK cell and CD8^+^ T cells are also observed in lung cancer (23, 24) where higher levels of granzyme B after immunotherapy furthermore correlate with better clinical outcome (25). The shift towards CD56^bright^ NK cells in CTCL skin could indicate that they are less cytotoxic and more cytokine secreting. In line with this, a smaller fraction of CTCL NK cells expressed the maturation marker CD57 and less mature NK cells are regarded as less cytotoxic (26). Somewhat surprising, CTCL skin NK cells expressed high levels of the activating receptor NKp46, known to be downregulated on NK cells in cancer (27). NK cells belong to the family of innate lymphoid cells (ILC), consisting of NK cells, ILC1s, ILC2s, ILC3s and lymphoid tissue inducer (LTi) cells, and definite markers for the identification of these subgroups of ILCs are lacking. NKp46 is a marker expressed by NK cells but can also be expressed by ILC1s and ILC3s (28). Hence, ILC1s and ILC3s could be within our pool of identified NK cells. A subset of monocytes and dendritic cells can also express CD56 (29–31), but because of the high expression of NKp46 of the skin derived CD3^-^CD56^+^ cells, these cell types could not constitute more than a very small minority of the analyzed cells. Nevertheless, increased expression of NKp46 on CTCL skin NK cells could indicate that CTCL NK cells can still be activated to induce tumor killing, and NK cell stimulation *ex vivo* or locally *in vivo* could thus represent promising future possibilities for immunotherapy in CTCL. The retained expression of CD16 and even upregulated expression of NKp46 on CTCL skin NK cells opens up for using trifunctional NK cells engagers targeting CD16, NKp46 and tumor antigen to elicit anti-tumor responses by NK cells (32). Alternatively, since CTCL skin NK cells could be activated to secrete IFNγ, NK cell activating cytokines represents another potential strategy for immunotherapy. For example, IL-15 super-agonists can block TGFβ-mediated inhibition of NK cells leading to NK cell tumor cell killing (33). However, even though IL-15 immunotherapy was shown to strongly increase the number of NK cells in malignancies, a mono-therapy with IL-15 was so far proven ineffective, probably due to counter acting immune-regulatory responses (34). Hence, combination therapies using IL-15 and inhibition of immune checkpoint proteins might be more effective (34).

Our results did not support exhaustion and/or upregulation of immune checkpoint proteins on NK and CD8^+^ T cells in CTCL compared to healthy cells. A higher proportion of CTCL CD8^+^ T cells were positive for PD1 compared to NK cells in both CTCL and healthy skin. We observed a small proportion of phenotypically exhausted NK cells in healthy skin, as indicated by PD1, NKG2A or CD73 expression. This could be the result of NK cell activation due to surveillance for tumor or otherwise stressed cells (35). These markers are furthermore not exclusively expressed by exhausted cells, as blood NK cells from healthy individuals can be positive for PD1, possibly as a result of cytomegalovirus infection (36). Anti-PD1 treatment was tested in a small clinical trial with 24 CTCL patients, and led to an overall response rate (ORR) of 38% with two complete responses and seven partial responses (37). Based on the low percentages of PD1 positive NK cells in CTCL skin in our study, lack of effect of PD1 inhibition on NK cells could partially explain the low response rate. Furthermore, single cell RNA sequencing revealed a high heterogeneity of tumor infiltrating CD8^+^ T cells in CTCL skin with a strong variation in the expression of co-inhibitory receptors PD1, CTLA4, TIM3, LAG3, and TIGIT (14), which could affect capacity of tumor cell killing. Recently, it was shown that NK cells can engage with malignant cells via 4-1BB resulting in the upregulation of the immune checkpoint protein CD73. CD73 positive NK cells can secrete TGFβ and IL-10, and thereby act as tumor promoters (38). Within our study, NK cells from fresh CTCL patient skin did not show higher levels of CD73 and had reduced IL-10 levels, suggesting that CTCL NK cells do not act in a tumor-promoting fashion.

Co-culture experiments of NK cells with the CTCL cell line HH induced a less active NK cell phenotype with reduced granzyme B levels as compared to cells co-culture with the non-malignant T cell line MyLa CD4. This is in line with the reduced granzyme B levels observed in primary CTCL skin NK cells and indicates that NK cells co-cultured with CTCL cells could have released granzyme B to kill malignant cells with reduction of cytotoxic capacity as a consequence. Furthermore, NK cells in close contact with non-malignant T cells could be activated as indicated by upregulation of CD69 and IFNγ, while NK cells in the environment of malignant HH cells failed to do so. Activated non-malignant CD4^+^ T cells can secrete IL2 that can trigger NK cells to release IFNγ (39). Hence, induction of IFNγ and CD69 by NK cells upon co-culture with non-malignant MyLa cells could be the effect of activating cytokines secreted by non-malignant MyLa cells. This is supported by the finding that conditioned medium of MyLa cells also significantly induced IFNγ in NK cells. The inability of malignant HH cells to activate NK cells could in a reversed manner be the result of a reduced secretion of activating cytokines or the secretion of blocking factors. Tumor cells are known to secrete immunosuppressive factors such as TGFβ, IL-10, adenosine, prostaglandine E2 (PGE2), idoleamine 2,3-dioxygenase (IDO) and lactate leading to tumor cell evasion (40, 41). One such blocking factor in CTCL could be TGFβ (18), which is known to inhibit the induction of granzyme B, IFNγ and perforin and the release of cytotoxic granules (42). In summary, co-culture of NK cells with malignant CTCL cells partially phenocopies the phenotype observed in fresh CTCL skin NK cells with reduced CD69 and granzyme B levels, suggesting that close contact with malignant cells contributes to this phenotype.

We could not identify significant differences between phenotypic markers expressed on CTCL skin NK cells and CD8^+^ T cells in patch *vs* plaque lesions, possibly due to the small study cohort or inter-individual variances in type of treatment, age or sex. Therefore, future investigations are needed with in-depth analysis of phenotypical markers of NK cells in a bigger study cohort by e.g. scRNA sequencing to identify biomarkers for patch and plaque that could help to predict disease progression and guide decisions on clinical monitoring and treatment.

The exact mechanisms behind the observed NK cell alterations in CTCL skin, as well as the possible clinical implications, still remain to be determined. One possible cause for downregulation of granzyme B might be the direct interactions between the NK cells and CTCL cells in the local microenvironment, underlining the importance to investigate anti-lymphoma immune responses at the site of the lymphoma.

## Data availability statement

The original contributions presented in the study are included in the article/**Supplementary Material.** Further inquiries can be directed to the corresponding author.

## Ethics statement

The studies involving humans were approved by Regionala etikprövningsnämnden i Stockholm, Sweden and Etikprövningsmyndigheten, Uppsala, Sweden. The studies were conducted in accordance with the local legislation and institutional requirements. The participants provided their written informed consent to participate in this study.

## Author contributions

Conceptualization and data curation: HB. Formal Analysis: AS, JN, MX, AW. Funding acquisition: HB. Investigation: AS, JN, MX, AW. Methodology: HB, AS. Project administration: HB. Resources: HB, LE, ME. Supervision: HB. Validation: AS, JN. Visualization: AS, JN. Writing – original draft: HB, AS. Writing – review & editing: all authors. All authors contributed to the article and approved the submitted version.
