# Supplementary material for: Skin infiltrating NK cells in cutaneous T-cell lymphoma are increased in number and display phenotypic alterations partially driven by the tumor
Source: Front Immunol. 2023 Aug 25;14:1168684. doi: 10.3389/fimmu.2023.1168684 (PMC10485839; doi:10.3389/fimmu.2023.1168684)
Supplement: Supplementary file 1 [file DataSheet_1.docx]

**Suppl Figures**


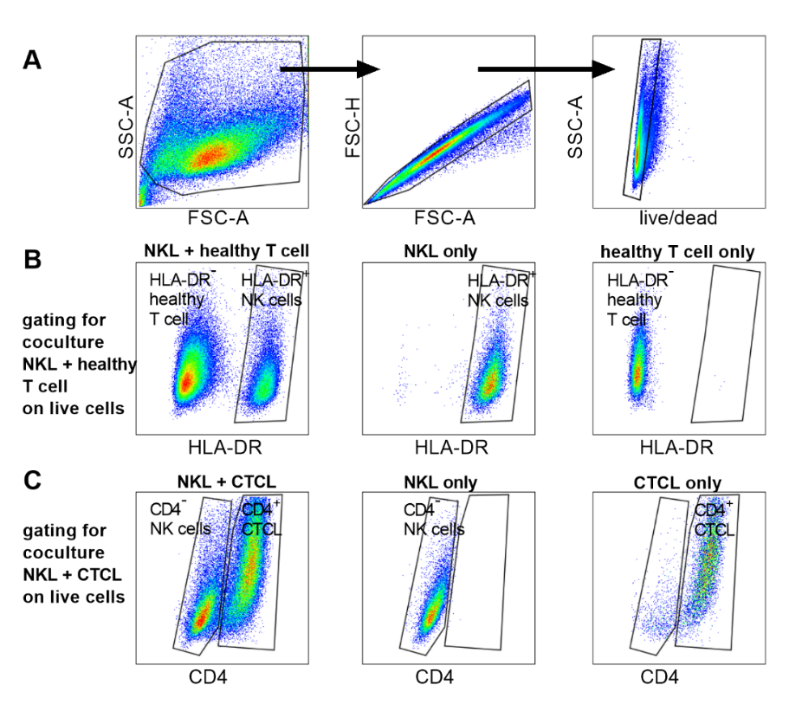


**Figure S1 = Gating strategy for coculture experiments of NKL cell line with healthy T cell line and CTCL cell line.** NKL cells and healthy T cells/ CTCL cells were cocultured and stained for flow cytometry analysis. **(A)** For the analysis, cell debris were excluded and cells were gated on single live cells. **(B)** Live NKL cells were discriminated from live healthy T cells using HLA-DR which is expressed by NKL cell line but not by the healthy T cell line. **(C)** Live NKL cells were discriminated from live CTCL cells using CD4 which is expressed by CTCL cells but not by NKL cells.

**
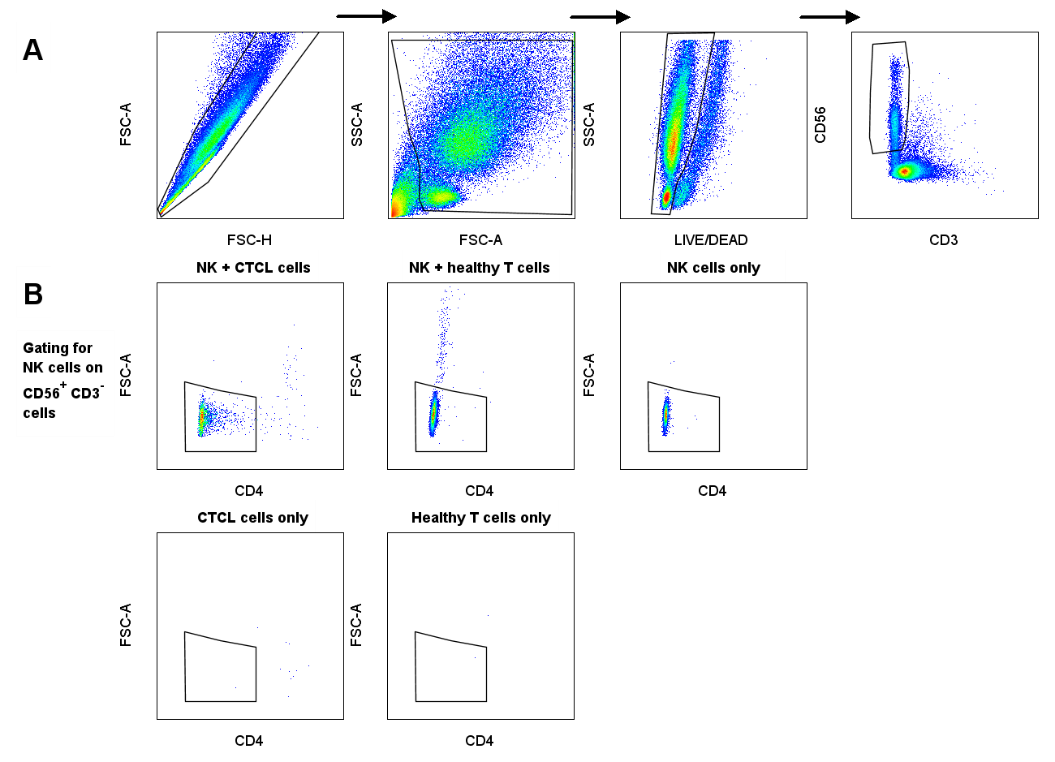
**

**Figure S2 = Gating strategy for coculture experiments of primary NK cells with healthy T cell line and CTCL cell line.** NK cells and CTCL cells/ healthy T cells were cocultured and stained for flow cytometry analysis. **(A)** For the analysis, cells were gated for single cells and cell debris were excluded. Live NK cells were discriminated from live CTCL cells/healthy T cells by gating for CD56^+^CD3^-^ cells. **(B)** The few remaining CTCL cells were excluded using CD4 which is expressed by CTCL cells but to a lesser extent by NK cells. The remaining healthy T cells were excluded based on their bigger size (FSC-A) compared to NK cells.

**
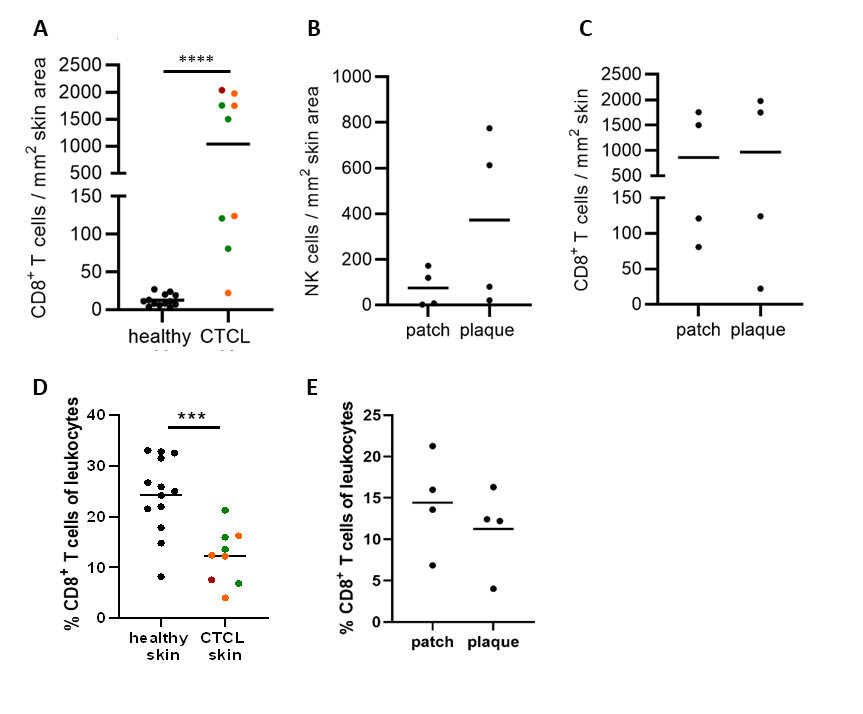
**

**Figure S3 = Numbers and percentages of CD8^+^ T cells in fresh patch and plaque CTCL and healthy skin as well as NK cells in fresh patch and plaque CTCL skin as identified by flow cytometry.** **(A)** CD8^+^ T cells/mm^2^ skin surface area. **(B)** NK cells/mm^2^ skin surface area in patch and plaque. **(C)** CD8^+^ T cells/mm^2^ skin surface area in patch and plaque. **(D)** CD8^+^ T cell percentages of total leukocytes. **(E)** CD8^+^ T cell percentages in patch and plaque. Cells were gated on singlet lymphocytes, live cells, CD45^+^ cells and thereafter CD56^+^CD3^-^ NK cells and CD3^+^CD8^+^ T cells. Data from fresh skin biopsies of thirteen healthy controls and eight CTCL patients (one patient contributed with two biopsies, one from a patch and one from a plaque lesion). Mann-Whitney U-tests have been performed. * = p-value < 0.05, ** = p-value < 0.01, *** = p-value < 0.001. **** = p-value < 0.001.


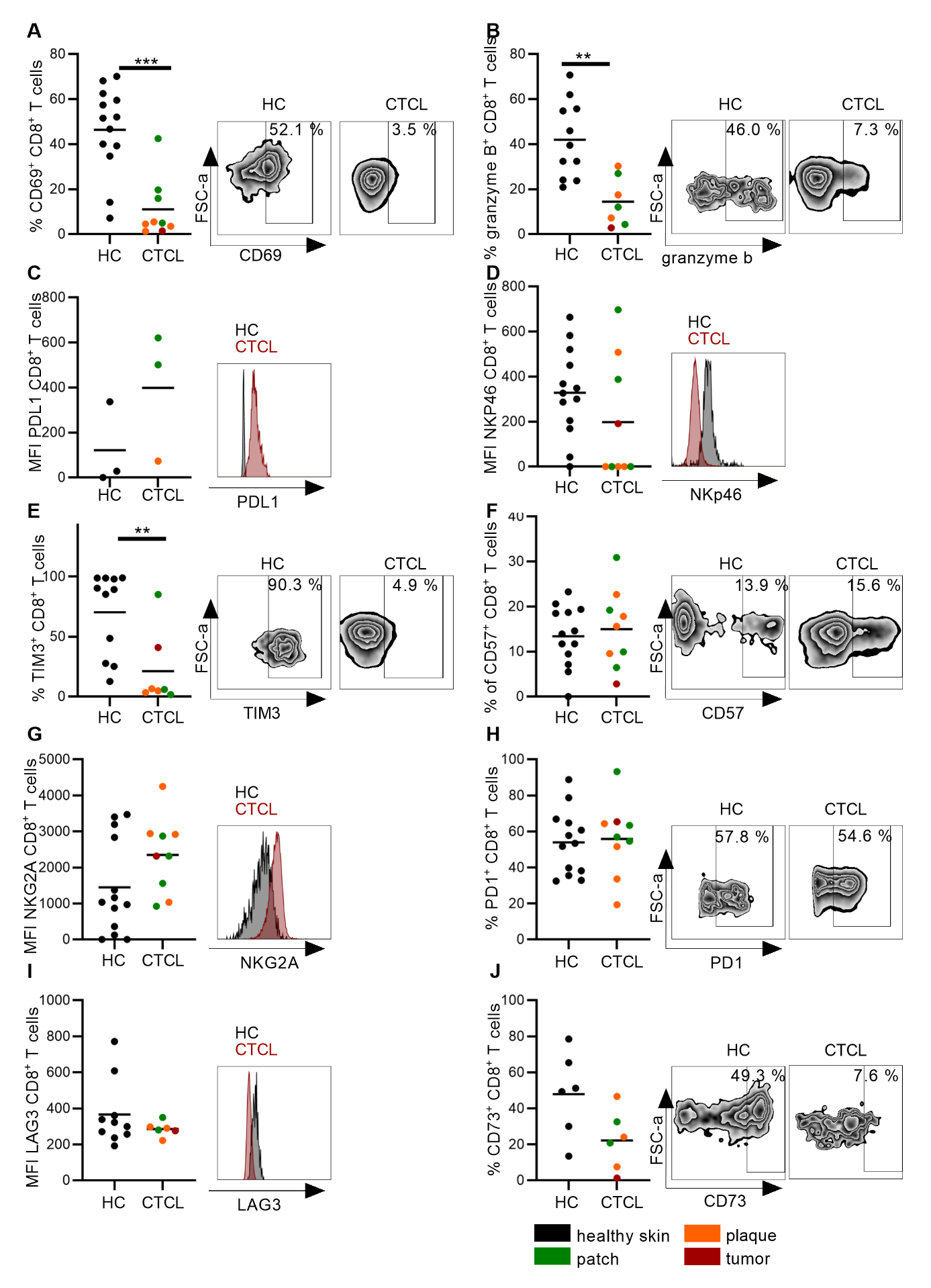


**Figure S4 = Distinct phenotype of CTCL skin CD8+ T cells indicates reduced activation.** CTCL CD8^+^ T cells and healthy skin derived CD8^+^ T cells were characterized by the early activation marker CD69 **(A),** the cytotoxic protein granzyme B **(B)**, the immune check point protein PDL1 **(C)**, the activating receptor NKp46 **(D)**, the immune checkpoint protein TIM3 **(E)**, the maturation marker CD57 **(F)**, the inhibitory receptor NKG2A **(G)** and the immune checkpoint proteins PD1 **(H)**, LAG3 **(I)** and CD73 **(J).** Cells were gated on singlet lymphocytes, live cells, CD45+ cells and thereafter CD3^+^CD8^+^ T cells. Data from fresh skin biopsies of thirteen healthy controls and eight CTCL patients (one patient contributed with two biopsies, one from a patch and one from a plaque lesion). Some phenotypic markers were not analyzed in all biopsies. Mann‑Whitney U‑tests were performed. * = p-value < 0.05, ** = p-value < 0.01, *** = p-value < 0.001.


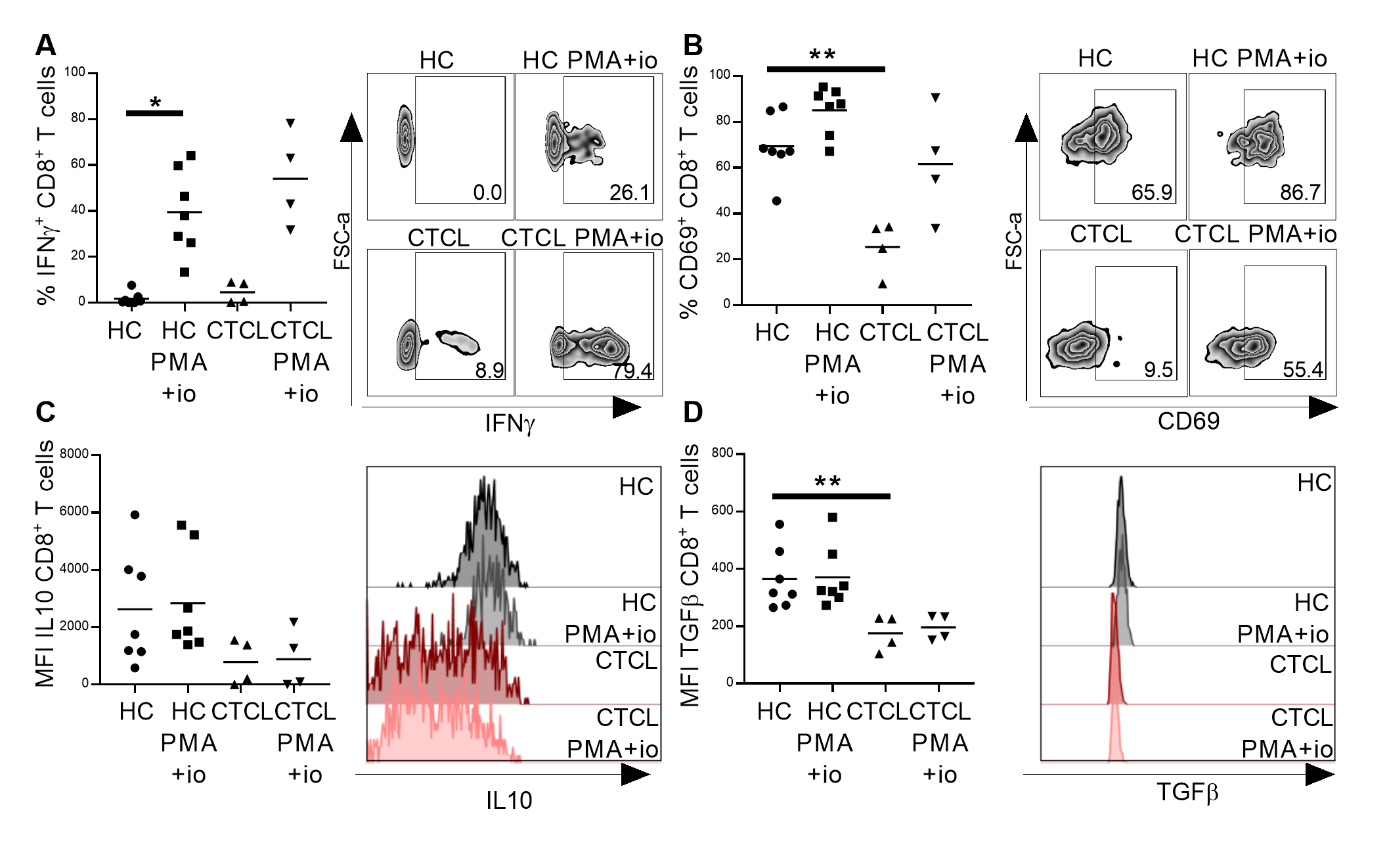


**Figure S5 =** **Primary CTCL skin CD8^+^ T cells are able to produce IFNγ upon activation with PMA and ionomycin in vitro.** Total lymphocytes extracted from CTCL and healthy skin were stimulated for 4 h with PMA and ionomycin followed by flow cytometry analysis of CD8^+^ T cells for IFNγ **(A)**, CD69 **(B)**, IL10 **(C)** and TGFβ **(D)**. Cells were gated on singlet lymphocytes, live cells, CD45^+^ cells and thereafter CD3^+^CD8^+^ T cells. Data from fresh skin biopsies of seven healthy controls and four CTCL patients. Mann-Whitney U-tests were performed to assess differences between healthy skin and CTCL skin. Wilcoxon tests were performed to assess differences between unstimulated samples and stimulated samples. * = p-value < 0.05, ** = p-value < 0.01, *** = p-value < 0.001.

**
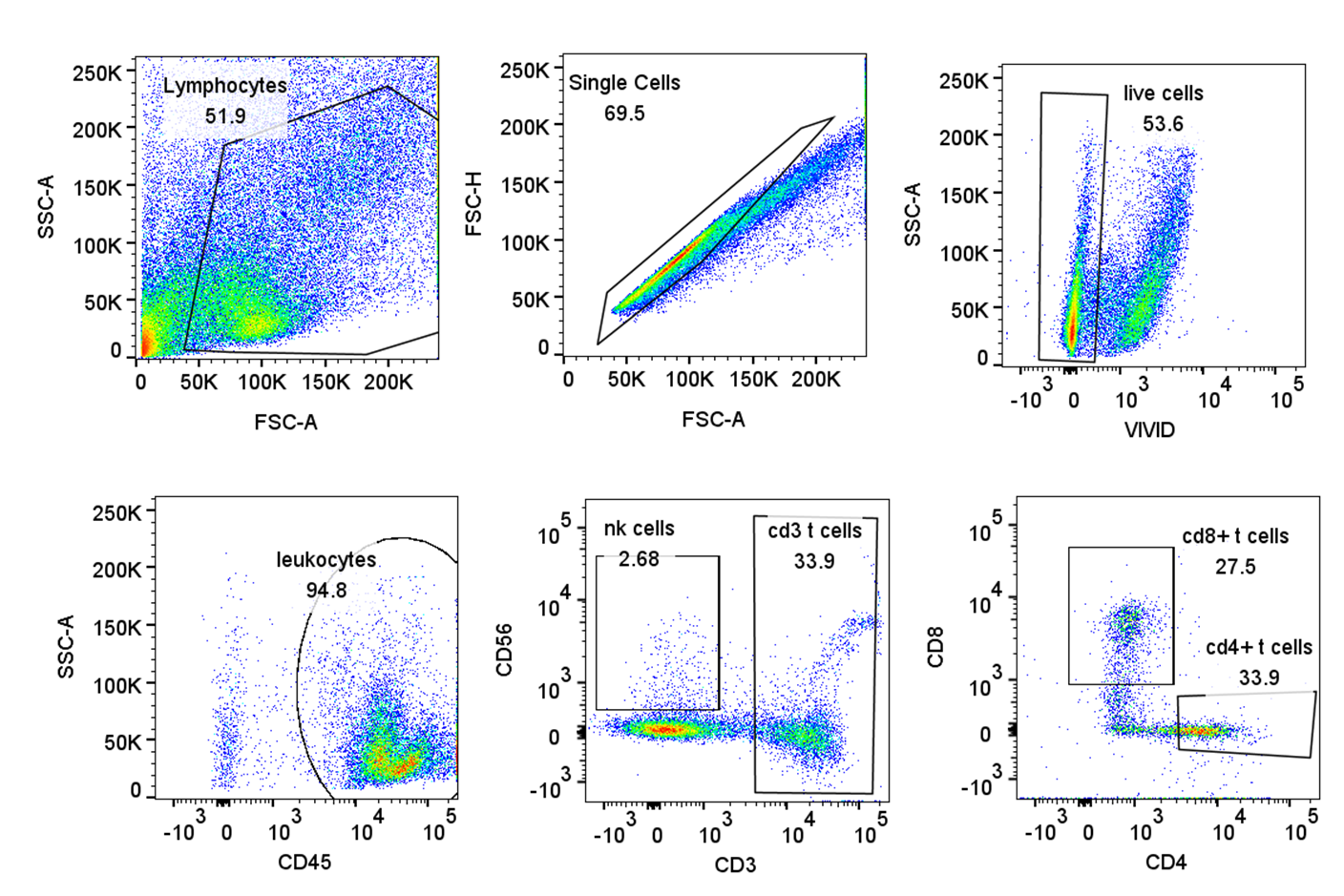
**

**Figure S6 = Gating strategy for PMA and ionomycin stimulation experiment.** Cells were gated on lymphocytes, singlet cells, live cells, CD45^+^ cells and thereafter CD56^+^CD3^-^ NK cells and CD3^+^CD8^+^ T cells.


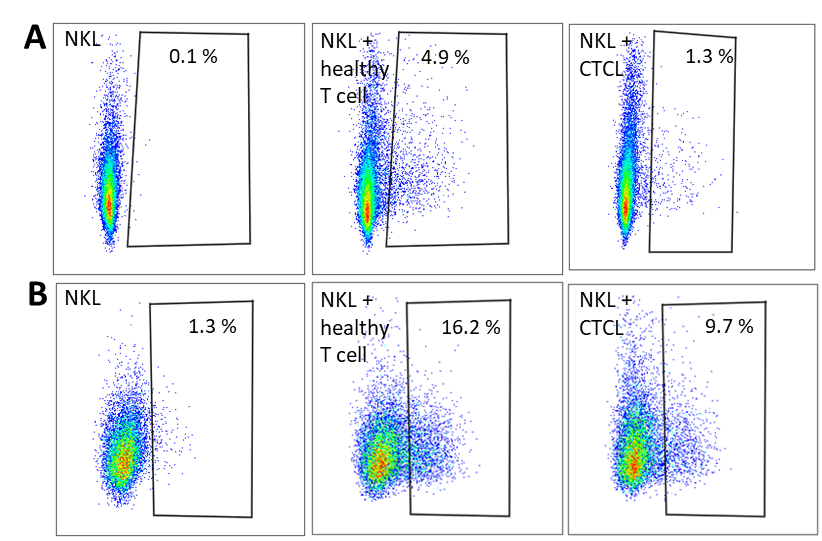


**Figure S7 = Dot plots representing IFNγ-positive and CD69-positive NKL cells.** NKL in monoculture, in Co-culture with healthy T cells and in Co-culture with the CTCL cell line have been gated for **(A)** IFN^+^ and **(B)** CD69^+^ NKL cells.

**Table S1. Antibodies used for IHC and Flox cytometry analysis.** Antibodies used for IHC and flow cytometry, the company, catalogue number, clone (when given by the company) and dilutions used.

| **Antibody** | **Company** | **cat. No.** | **Clone** | **Dilution used** |
| --- | --- | --- | --- | --- |
| mouse anti-CD56 (monoclonal)) | Dako/Agilent | M730429-2 | clone 123C3 | 1:400 |
| rat anti-CD3 (monoclonal) | Abcam | ab11089 | CD3-12 | 1:200 |
| rabbit anti-CD8 (polyclonal) | Abcam | ab4055 | -- | 1:100 |
| goat anti-mouse IgG H&L HRP (polyclonal) | Abcam | ab97040 | -- | 1:200 |
| goat anti-rat IgG NorthernLights^TM^ 637 (polyclonal) | R&D system | NL014 | -- | 1:100 |
| donkey anti-rabbit IgG (H+L) Alexa Flour^TM^ 594 (polyclonal) | Life Technologies | #21207 | -- | 1:1000 |
| anti-CD3 PE/Cy5 | Nordic Biosite | 300310 | HIT3a | 1:28 |
| anti-CD4 BUV395 | BD Biosciences | 563550 | SK3 | 1:40 |
| anti-CD8 APC/Cy7 | Nordic Biosite | 344714 | SK1 | 1:14 |
| anti-CD16 PerCP | Nordic Biosite | 30-2449-100 | 3G | 1:28 |
| anti-CD45 BV650 | BD Biosciences | 563717 | HI30 | 1:14 |
| anti-CD56 PE/Cy7 | Nordic Biosite | 318318 | HCD56 | 1:28 |
| anti-CD57 BV605 | Nordic Biosite | 393304 | QA17A04 | 1:28 |
| anti-CD69 BV711 | BD Biosciences | 563836 | FN50 | 1:28 |
| anti-CD73 BUV737 | BD Biosciences | 612813 | AD2 | 1:14 |
| anti-TIM3 APC | Nordic Biosite | 345012 | F38-2E2 | 1:14 |
| anti-PD1 PE | Nordic Biosite | 329906 | EH12.2H7 | 1:14 |
| anti-NKG2A BV786 | BD Biosciences | 747917 | 131411 | 1:10 |
| anti-NKp46 PE/Dazzle 594 | Nordic Biosite | 331930 | 9E2 | 1:28 |
| anti-CD107a PE/Cy7 | Nordic Biosite | 328618 | H4A3 | 1:100 |
| anti-CD107a PerCP | Nordic Biosite | 328642 | H4A3 | 1:100 |
| anti-TGFβ Alexa Fluor 488 | BD Biosciences | 562545 | TW4-9E7 | 1:20 |
| anti-IL 10 APC | BD Biosciences | 554707 | JES3-19F1 | 1:20 |
| anti-granzyme B BV421 | BD Biosciences | 563389 | GB11 | 1:40 |
| anti-IFNγ PE | BD Biosciences | 554552 | 4S.B3 | 1:20 |
